# Supplementary material for: Modeling glioblastoma heterogeneity as a dynamic network of cell states
Source: Mol Syst Biol. 2021 Sep 16;17(9):e10105. doi: 10.15252/msb.202010105 (PMC8444284; doi:10.15252/msb.202010105)
Supplement: Supplementary file 6 — Source Data for Figure 5 [file MSB-17-e10105-s004.zip › Figure5A_sourcedata/GSEA_3071/hallmarks_stateVI.GseaPreranked.1623742557031/HALLMARK_KRAS_SIGNALING_UP.html]

Details for gene set HALLMARK\_KRAS\_SIGNALING\_UP[GSEA]

|  || Dataset | state63071 |
| Phenotype | NoPhenotypeAvailable |
| Upregulated in class | na\_neg |
| GeneSet | HALLMARK\_KRAS\_SIGNALING\_UP |
| Enrichment Score (ES) | -0.3535164 |
| Normalized Enrichment Score (NES) | -1.5860121 |
| Nominal p-value | 0.03165299 |
| FDR q-value | 0.1448364 |
| FWER p-Value | 0.778 |
Table: GSEA Results Summary

  

Fig 1: Enrichment plot: HALLMARK\_KRAS\_SIGNALING\_UP      
 Profile of the Running ES Score & Positions of GeneSet Members on the Rank Ordered List

  

| PROBE | GENE SYMBOL | GENE\_TITLE | RANK IN GENE LIST | RANK METRIC SCORE | RUNNING ES | CORE ENRICHMENT || 1 | DCBLD2 |  |  | 16 | 1.066 | 0.0262 | No |
| 2 | F13A1 |  |  | 23 | 0.999 | 0.0532 | No |
| 3 | PLAUR |  |  | 34 | 0.936 | 0.0774 | No |
| 4 | ITGA2 |  |  | 71 | 0.810 | 0.0906 | No |
| 5 | INHBA |  |  | 80 | 0.781 | 0.1108 | No |
| 6 | AKAP12 |  |  | 92 | 0.743 | 0.1291 | No |
| 7 | PPP1R15A |  |  | 98 | 0.738 | 0.1489 | No |
| 8 | HBEGF |  |  | 172 | 0.627 | 0.1466 | No |
| 9 | BIRC3 |  |  | 191 | 0.608 | 0.1591 | No |
| 10 | PTPRR |  |  | 207 | 0.598 | 0.1721 | No |
| 11 | GLRX |  |  | 330 | 0.523 | 0.1531 | No |
| 12 | GFPT2 |  |  | 436 | 0.476 | 0.1374 | No |
| 13 | TNFAIP3 |  |  | 445 | 0.473 | 0.1488 | No |
| 14 | YRDC |  |  | 674 | 0.405 | 0.0969 | No |
| 15 | IL7R |  |  | 718 | 0.392 | 0.0962 | No |
| 16 | PRELID3B |  |  | 892 | 0.361 | 0.0583 | No |
| 17 | BMP2 |  |  | 940 | 0.354 | 0.0554 | No |
| 18 | PTCD2 |  |  | 959 | 0.352 | 0.0604 | No |
| 19 | TRAF1 |  |  | 1040 | 0.339 | 0.0479 | No |
| 20 | CCND2 |  |  | 1064 | 0.335 | 0.0511 | No |
| 21 | TSPAN13 |  |  | 1195 | 0.317 | 0.0239 | No |
| 22 | TFPI |  |  | 1205 | 0.315 | 0.0305 | No |
| 23 | ITGBL1 |  |  | 1398 | 0.292 | -0.0147 | No |
| 24 | TMEM158 |  |  | 1466 | 0.283 | -0.0253 | No |
| 25 | DNMBP |  |  | 1596 | 0.269 | -0.0535 | No |
| 26 | AMMECR1 |  |  | 1726 | 0.258 | -0.0821 | No |
| 27 | LIF |  |  | 1804 | 0.250 | -0.0964 | No |
| 28 | ADAM17 |  |  | 1855 | -0.258 | -0.1029 | No |
| 29 | MYCN |  |  | 1860 | -0.259 | -0.0966 | No |
| 30 | CPE |  |  | 2173 | -0.309 | -0.1747 | No |
| 31 | HKDC1 |  |  | 2527 | -0.384 | -0.2622 | No |
| 32 | STRN |  |  | 2546 | -0.387 | -0.2561 | No |
| 33 | SPRY2 |  |  | 2569 | -0.391 | -0.2509 | No |
| 34 | TSPAN7 |  |  | 2848 | -0.475 | -0.3148 | No |
| 35 | NIN |  |  | 2891 | -0.491 | -0.3124 | No |
| 36 | SATB1 |  |  | 2899 | -0.494 | -0.3002 | No |
| 37 | ID2 |  |  | 3026 | -0.554 | -0.3194 | No |
| 38 | GNG11 |  |  | 3137 | -0.612 | -0.3325 | Yes |
| 39 | GYPC |  |  | 3169 | -0.638 | -0.3228 | Yes |
| 40 | SOX9 |  |  | 3280 | -0.739 | -0.3323 | Yes |
| 41 | PRRX1 |  |  | 3288 | -0.749 | -0.3127 | Yes |
| 42 | CXCR4 |  |  | 3302 | -0.759 | -0.2945 | Yes |
| 43 | RGS16 |  |  | 3304 | -0.765 | -0.2728 | Yes |
| 44 | KIF5C |  |  | 3354 | -0.821 | -0.2629 | Yes |
| 45 | ETV1 |  |  | 3378 | -0.857 | -0.2446 | Yes |
| 46 | PLAT |  |  | 3416 | -0.927 | -0.2283 | Yes |
| 47 | GPRC5B |  |  | 3459 | -1.026 | -0.2105 | Yes |
| 48 | ETV5 |  |  | 3466 | -1.043 | -0.1822 | Yes |
| 49 | TRIB2 |  |  | 3488 | -1.088 | -0.1568 | Yes |
| 50 | DUSP6 |  |  | 3512 | -1.151 | -0.1301 | Yes |
| 51 | PCSK1N |  |  | 3522 | -1.197 | -0.0982 | Yes |
| 52 | SPP1 |  |  | 3541 | -1.286 | -0.0663 | Yes |
| 53 | MAP3K1 |  |  | 3584 | -1.605 | -0.0319 | Yes |
| 54 | IGFBP3 |  |  | 3588 | -1.623 | 0.0139 | Yes |
Table: GSEA details [plain text format]

  

Fig 2: HALLMARK\_KRAS\_SIGNALING\_UP: Random ES distribution      
 Gene set null distribution of ES for **HALLMARK\_KRAS\_SIGNALING\_UP**
